# Effects of intensity of electroacupuncture on chronic pain in patients with knee osteoarthritis: a randomized controlled trial

**DOI:** 10.1186/s13075-019-1899-6

**Published:** 2019-05-14

**Authors:** Zheng-tao Lv, Lin-lin Shen, Bing Zhu, Zhao-qing Zhang, Chao-yang Ma, Guo-fu Huang, Jing Yin, Ling-ling Yu, Si-yi Yu, Ming-qiao Ding, Jing Li, Xiao-cui Yuan, Wei He, Xiang-hong Jing, Man Li

**Affiliations:** 1 Department of Neurobiology, School of Basic Medicine, Tongji Medical College of Huazhong University of Science and Technology, Wuhan, 430030 China; 2 Department of Orthopedics, Tongji Hospital, Tongji Medical College of Huazhong University of Science and Technology, Wuhan, 430030 China; 3 Combined Traditional Chinese and Western Medicine Hospital affiliated to Tongji Medical College of Huazhong University of Science and Technology, Wuhan, 430030 China; 4 Institute of Acupuncture and Moxibustion, China Academy of Chinese Medical Sciences, Beijing, 100700 China; 5 The Third Hospital of Wuhan, Wuhan, 430060 China; 6 Central Hospital of Wuhan, Wuhan, 430014 China; 7 School of Acupuncture and Moxibustion, Chengdu University of Traditional Chinese Medicine, Chengdu, 610075 China; 8 The Fifth Hospital of Wuhan, Wuhan, 430050 China; 9 Union Hospital affiliated to Tongji Medical College of Huazhong University of Science and Technology, Wuhan, 430022 China

**Keywords:** Electroacupuncture, Knee osteoarthritis, Diffuse noxious inhibitory control, Randomized controlled trial, Pain

## Abstract

**Background:**

Conditioned pain modulation (CPM) is impaired in people with chronic pain such as knee osteoarthritis (KOA). The purpose of this randomized, controlled clinical trial was to investigate whether strong electroacupuncture (EA) was more effective on chronic pain by strengthening the CPM function than weak EA or sham EA in patients with KOA.

**Methods:**

In this multicenter, three-arm parallel, single-blind randomized controlled trial, 301 patients with KOA were randomly assigned. Patients were randomized into three groups based on EA current intensity: strong EA (> 2 mA), weak EA (< 0.5 mA), and sham EA (non-acupoint). Treatments consisted of five sessions per week, for 2 weeks. Primary outcome measures were visual analog scale (VAS), CPM function, and Western Ontario and McMaster Universities Osteoarthritis Index (WOMAC).

**Results:**

Three hundred one patients with KOA were randomly assigned, among which 271 (90.0%) completed the study (mean age 63.93 years old). One week of EA had a clinically important improvement in VAS and WOMAC but not in CPM function. After 2 weeks treatment, EA improved VAS, CPM, and WOMAC compared with baseline. Compared with sham EA, weak EA (3.8; 95% CI 3.45, 4.15; *P* < .01) and strong EA (13.54; 95% CI 13.23, 13.85; *P* < .01) were better in improving CPM function. Compared with weak EA, strong EA was better in enhancing CPM function (9.73; 95% CI 9.44, 10.02; *P* < .01), as well as in reducing VAS and total WOMAC score.

**Conclusion:**

EA should be administered for at least 2 weeks to exert a clinically important effect on improving CPM function of KOA patients. Strong EA is better than weak or sham EA in alleviating pain intensity and inhibiting chronic pain.

**Trial registration:**

This study was registered with the Chinese Clinical Trial Registry (ChiCTR-ICR-14005411), registered on 31 October 2014.

**Electronic supplementary material:**

The online version of this article (10.1186/s13075-019-1899-6) contains supplementary material, which is available to authorized users.

## Background

Acupuncture is an ancient therapeutic technique for pain treatment, which has been proved to have a promising analgesic effect on chronic pain disorders in clinical studies [1, 2]. Electroacupuncture (EA), an important form of acupuncture, has been widely used as a substitute for classical acupuncture [3, 4]. Knee osteoarthritis (KOA) is a common and disabling condition that typically manifests as attacks of pain around the joints, and it is a typical disease which can develop chronic pain [5]. Both acupuncture and EA have been shown to be effective in the treatment of chronic pain of KOA in randomized controlled trials (RCT) [1, 6, 7]. It has been demonstrated that the intensity of EA is very important for its analgesic effect in animal experiments [8–10]. Moreover, Barlas Panos found that high intensity of EA is more effective in relieving experimental pain in healthy human volunteers than low intensity of EA in RCT [11]. However, the effects of high or low intensity of EA (strong or weak EA) on chronic pain in patients with KOA remain unknown.

The concept of conditioned pain modulation (CPM), previously referred to as diffuse noxious inhibitory controls (DNIC), indicates that under normal conditions, pain can be attenuated by conditioning to a remote body region [12]. The endogenous analgesic system is critical for handling noxious events, and the strength of CPM function can predict the potential of developing chronic pain [13–15]. Quante and colleagues reported that neuronal plasticity of the descending pain inhibitory system impacts CPM function, which is diminished during the development of KOA [16]. Previous study has demonstrated that high intensity of EA (> 2 mA) is similar to a noxious stimulus and may activate CPM function effectively in rats [10]. Thus, we hypothesized that high intensity of EA (strong EA) may be more effective on chronic pain in patients with KOA by strengthening the CPM function. To validate this hypothesis, we undertook this randomized controlled trial to compare the effect of strong EA with weak EA or sham EA on chronic pain in patients with KOA.

## Methods

### Ethics approval

The protocol of this clinical trial was in adherence to the STRICTA guidelines and has been described in detail elsewhere [17]. This study was approved by the Chinese Ethics Committee of Registering Clinical Trials (reference: ChiECRCT-20140035) and registered with the Chinese Clinical Trial Registry (ChiCTR-ICR-14005411) on 31 October 2014 (http://www.chictr.org.cn/showproj.aspx?proj=9758). All patients provided written informed consent before randomization.

### Study design

This was a multicenter, three-arm parallel, randomized controlled trial to compare the efficacy of the two groups of EA (weak EA and strong EA) with sham EA. Patients were enlisted through hospital-based recruitment and advertisements with posters, leaflets, and newspapers from November 2014 to March 2016.

### Randomization and masking

After a 2-week washout period, patients who met our inclusion criteria were randomly assigned to one of the three groups (strong EA, weak EA, or sham EA) in a ratio of 2:1:1 using a computer-generated random allocation sequence through stratified block randomization method of SAS version 9.1.3 (SAS Institute, Cary, NC, USA). In our preliminary trial, we found that the effect of strong EA was better than that of weak EA. Moreover, the previous study investigating the efficacy of acupuncture compared with minimal acupuncture and no acupuncture in patients with KOA also used such a 2:1:1 randomization ratio [1]. For the consideration of patients’ welfare, the randomized group method was changed from 1:1:1 to 2:1:1. The randomization ratio in the register link in the Chinese Clinical Trial Registry (http://www.chictr.org.cn/showproj.aspx?proj=9758) has also been modified.

Randomization was performed by an independent research assistant who did not participate in any other section of this study. The acupuncturists were informed of the treatment assignment by mobile phone confirmation, and allocation concealment was not revealed until the final outcome analysis was reported.

Enrolled participants were only informed that they would receive one of the three acupuncture therapies; consequently, they were not aware of their treatment allocation. Acupuncturists were permitted to treat both knees if the two knees were affected by osteoarthritis. However, only the most symptomatic knee was evaluated for the outcome assessment throughout the study. Participants, clinical outcome evaluators, and statisticians were blinded to randomization, since it was not feasible to blind the acupuncturists who administered EA.

### Participants

A total of 450 patients with KOA were recruited from 5 hospitals in Wuhan, China: the Combined Traditional Chinese and Western Medicine Hospital affiliated to Tongji Medical College of Huazhong University of Science and Technology; the Third Hospital of Wuhan; Central Hospital of Wuhan; Union Hospital affiliated to Tongji Medical College of Huazhong University of Science and Technology; and the Fifth Hospital of Wuhan. People aged 50 years or older who met the clinical criteria for KOA formulated by the American College of Rheumatology (ACR) were deemed eligible for inclusion [18]. We excluded patients who had ever experienced adverse reactions to acupuncture prior to our study; who had comorbidities including severe cardiovascular, cerebral, hepatic, renal, or hematopoietic diseases; who had other disorders that might affect the knee (e.g., rheumatoid arthritis, gouty arthritis); who were pregnant or attempting to become pregnant or were lactating; and who had a history of mental illness. All participants were required not to take analgesic medications and EA 48 h before each treatment session.

### Interventions

Study interventions were performed by acupuncturists with at least 3 years clinical experience who were licensed Chinese medicine practitioners. To ensure standardization of the treatment protocol, each acupuncturist from the five hospitals underwent pretrial training on the study protocol, completing the case report form, treatment technique, and outcome assessment from the lead investigators who also monitored the process for this clinical study. Treatments for both the true EA groups and sham EA group consisted of ten 30-min sessions over 2 weeks. Assessments of participants were performed at baseline and at the end of the first and second weeks of the treatment phase.

During the 30-min stimulation period, participants were in a supine position with a pillow under each knee for support. Sterile disposable needles (30 gauge with an outer diameter of 0.32 mm and a length of 40 mm; Hwato, Suzhou, China) were used. Participants in the strong and weak EA groups received treatment at the same four acupoints of *Neixiyan* (EX-LE 5), *Dubi* (ST 35), *Liangqiu* (ST 34), and *Xuehai* (SP 10) unilaterally based on traditional Chinese medicine meridian theory [1, 19]. After local disinfection, needles were inserted to a depth of 25 to 40 mm vertically. *De qi* sensation was elicited by lifting and thrusting combined with twirling and rotating the needles. (*De qi* is the feeling experienced by patients at the needling site that includes fullness, heaviness, dull aching, or warmth and is indicative of effective needling.) Electrical stimulation was then applied using an EA apparatus (Shanghai Medical Electronic Instrument), with a pair of electrodes connecting acupoints EX-LE 5 with ST 35, and another pair of electrodes connecting SP 10 with ST 34 [20, 21]. Stimulation parameters were direct current, continuous wave, 2 Hz frequency, and 0.5 ms pulse width, for 30 min. After obtaining *de qi* sensation, the strong EA and weak EA groups received different stimulation intensity. The strong EA group received the maximum tolerable intensity of current between 2 and 5 mA. The weak EA group received low-intensity current between 0 and 0.5 mA. Once the current was felt, the participant informed the acupuncturist to stop increasing the current.

In the sham EA group, the number of acupoints, EA apparatus, and stimulation parameters were the same as for the true EA groups. However, the needles used in the sham EA group were fine and short (35-gauge needle with an outer diameter of 0.20 mm and a length of 25 mm; Hwato, Suzhou, China). The needles were inserted only superficially into non-acupoint sites, each 2 cm lateral to each of the four acupoints to an approximate depth of 5 to 10 mm. In addition, the needles were not manipulated to avoid obtaining *de qi* sensation. Electrical stimulation was delivered with the same low intensity as the weak EA group. For all three groups, after each session, all the needles and the EA electrodes were removed.

### Outcome measurements

Participants completed questionnaires before treatment, after 1 week and after 2 weeks. Primary outcome measures were pain visual analog scale (VAS), CPM value, and Chinese translations of the Western Ontario and McMaster Universities Osteoarthritis Index (WOMAC) [15, 22–25].

The VAS used in this study was a 10-cm line ranging from 0 (no pain) to 10 (pain as bad as it could be) that assessed peak pain intensity over the last 24 h. The WOMAC index consists of three domains, namely pain (5 items), stiffness (2 items), and physical function (17 items), and each item is scored based on a 5-point Likert numerable rating scale representing different degrees of intensity (none, mild, moderate, severe, or extreme). The final score of WOMAC was determined by adding the aggregate scores for three subscales, which ranges from 0 to 96, and a greater score indicates greater pain and dysfunction. The WOMAC has been translated and validated in Chinese [25].

In our current study, we used the terminology “CPM” instead of “DNIC,” because DNIC is a terminology used on animals. CPM represents the descending inhibitory modulation of pain. It can be assessed when two painful stimuli are applied simultaneously, the “conditioning” stimulus that typically inhibits the “test” stimulus [22]. In this study, to measure CPM, the acupuncturist applied a 180-g von Frey filament to the *Ashi* point (pain spot) of the affected knee within a 1-cm-diameter circle for three to five times. The participant was then asked to mark the intensity of pain on the VAS after each punch [15]. The research assistants recorded the mean value of the three VAS scores as VAS1. Next, the participant was asked to immerse the contralateral hand and wrist into cold water (10 to 12 °C) for 1 min. Afterward, the mean VAS score of pain elicited by the von Frey filament on the same *Ashi* point was recorded as VAS2. VAS scores were measured immediately after the conditioning stimulus since the CPM effects are generally short-lived. Percent changes were calculated for the change in CPM based on the following formula: CPM = (VAS1 − VAS2)/VAS1 × 100% [23, 24], where 0 indicated no change and higher values indicated more effective pain inhibition.

Secondary outcomes included the numeric pain rating scale (NPRS) [26], emotional scale (ES) [27], and present pain intensity (PPI) [28]. The research assistants assisted the participants in completing the survey instruments at the end of the 2-week study.

Research assistant documented severe adverse events and side effects associated with EA treatment. Participants were also asked to report side effects at the end of the study. Officers from the Scientific Research Office in the five hospitals formed the Data and Safety Monitoring Board, who periodically reviewed and evaluated the accumulated study data for participant safety, study conduct, and progress.

### Sample size calculation and statistical analysis

Sample size estimation was performed to detect a minimal clinically important difference (MCID) of 1.8 units in VAS pain score (extrapolated from 18 mm MCID reported for 100 mm VAS) [29]. We also aimed to detect an MCID of 6.7 units of total WOMAC score identified as the MCID for osteoarthritis (extrapolated from 7 units of MCID reported for 0–100 normalized WOMAC total score) [30]. However, no literature reports MCID of CPM in patients with KOA. So, we calculate the sample size based on VAS and WOMAC score. The calculated sample size based on at least 80% power, 15% drop out, and a two-sided 5% significance level gave a required population of 67 subjects. Seventy-five participants in each group will give us up to 95% power to detect the true effect.

The statistical analysis plan was completed and approved by the data and safety monitoring board. Analyses of the baseline characteristics and clinical outcomes were based on the intention-to-treat (ITT) population, which included participants who had been randomized and baseline data recorded (*n* = 292). Baseline characteristics were presented as percentages for categorical variables and mean (SD) with 95% CIs for continuous variables. Missing data were imputed for each group separately, using chained equations with predictive mean matching. A total of 20 imputed datasets were completed based on the raw dataset, and then corresponding estimates were combined using Rubin’s rules.

Multiple linear regressions were used to compare the significant differences in mean changes from baseline between the groups for each outcome and were adjusted for basic characteristics (sex, age, duration of KOA, and body mass index) and baseline outcome score. We conducted a between-group comparison of strong EA and weak EA with sham EA as a control in the linear model, as well as comparison of the strong EA and weak EA groups. Mixed effect model was used to test the significance of change of effect between any two time points in different treatment groups. All analyses were performed using R (version 3.2.4; The R Foundation, Vienna, Austria) and its mice package, with differences considered significant if the two-tailed *P* value was less than 0.05.

## Results

### Participants and baseline characteristics

**Fig. 1:**
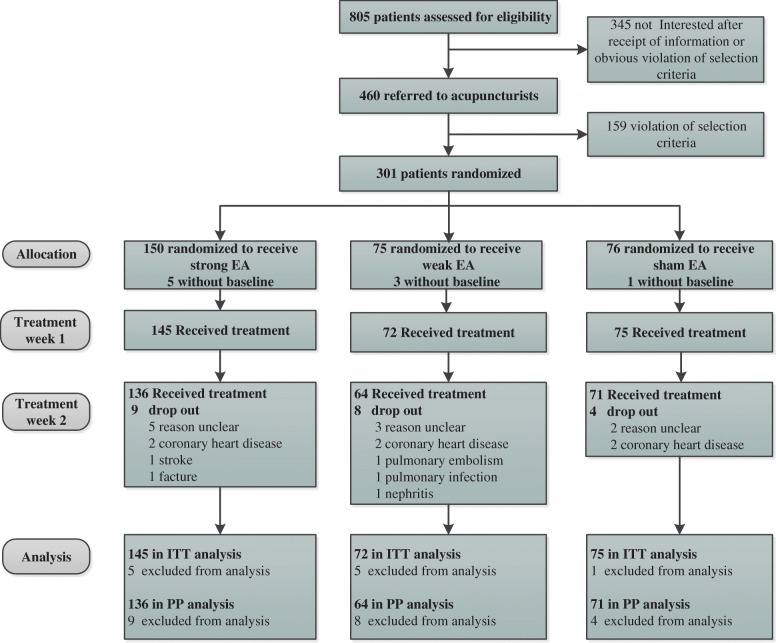
Trial flow chart

Between September 2014 and March 2016, we screened 805 participants for eligibility among the 5 hospitals, of whom 301 were randomly assigned. Among the randomized participants, 271 (90.0%) completed the study. Multiple imputations were used for the missing data in 21 participants (9 in the strong EA group, 8 in the weak EA group, and 4 in the sham EA group). Dropouts at each stage and the number assessed for the primary end point are presented in Fig. 1. The baseline characteristics of the participants are shown in Table 1. They were comparable across the 3 groups.

**Table 1:** Baseline characteristics of participants

Variable	Strong EA (*n* = 145)	Weak EA (*n* = 72)	Sham EA (*n* = 75)
Female, no. (%)	106 (73.1)	57 (79.2)	60 (80.0)
Age, mean (SD), years	64.6 (10.2)	63.7 (9.3)	61.9 (9.5)
Symptom duration, years (%)			
< 0.5 years	51 (35.2)	27 (37.5)	27 (36.0)
0.5 to 3 years	60 (41.4)	27 (37.5)	23 (30.7)
3 to 5 years	14 (9.7)	8 (11.1)	15 (20.0)
≥ 5 years	20 (13.8)	10 (13.9)	10 (13.3)
Height, mean (SD), m	1.62 (0.07)	1.62 (0.07)	1.64 (0.08)
Weight, mean (SD), kg	59.8 (7.8)	59.4 (7.5)	61.8 (8.5)
BMI, mean (SD)	22.67 (2.22)	22.46 (1.87)	22.93 (1.86)
Previous treatment, no. of patients (%)			
Physical therapy	25 (17.2)	11 (15.3)	12 (16.0)
Corticosteroid injections	5 (3.5)	2 (2.8)	2 (2.7)
Acupuncture	21 (14.5)	8 (10.8)	10 (13.3)
Exercise	36 (24.8)	15 (20.3)	16 (21.3)

*BMI* body mass index

### Primary and secondary outcomes

**Fig. 2:**
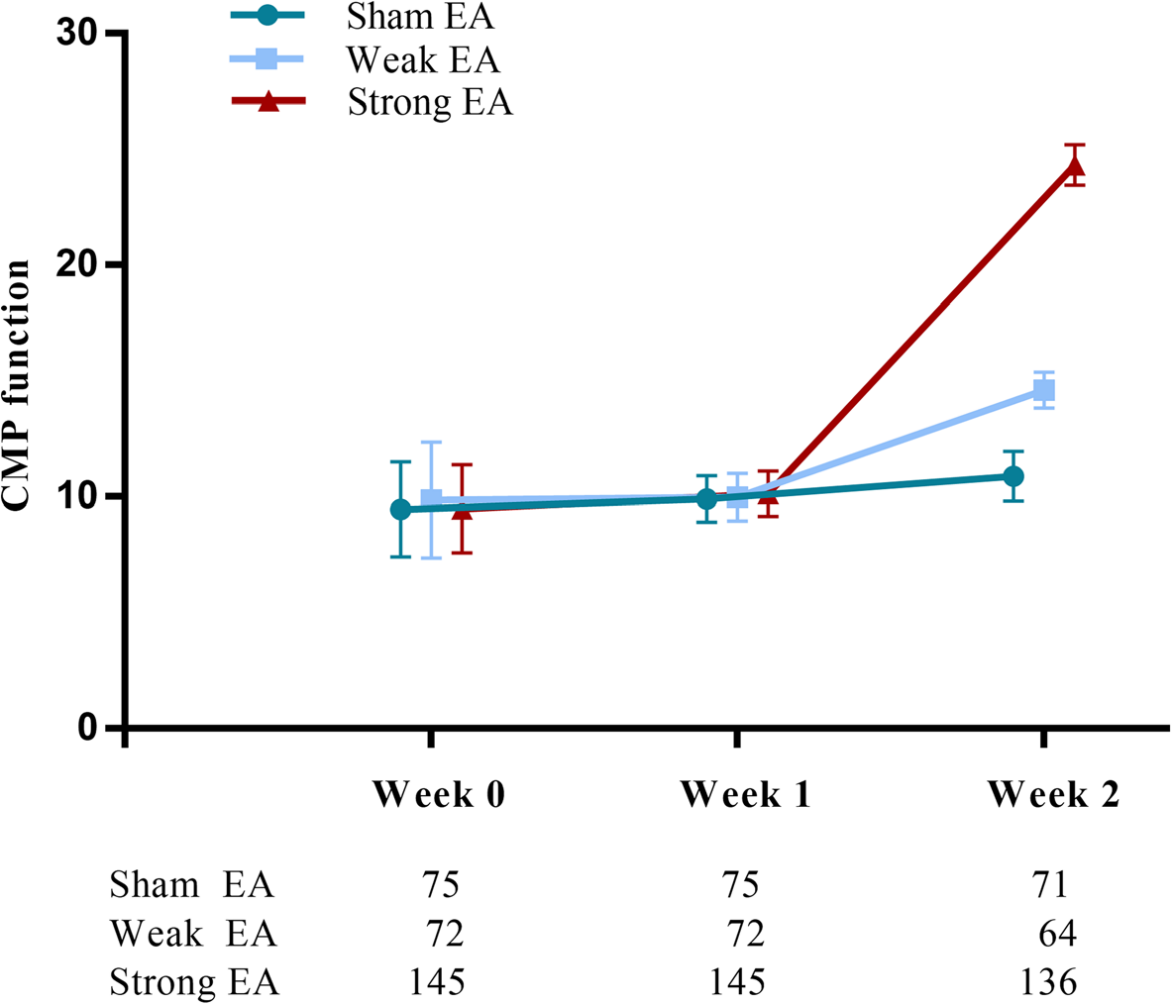
Mean CPM scores in sham, weak, and strong EA groups over 2 weeks treatment

For the primary outcome, the mean of CPM function score was 9.49 at baseline and 24.34 at week 2 in the strong EA group, 9.86 at baseline and 14.61 at week 2 in the weak EA group, and 9.46 at baseline and 10.89 at week 2 in the sham EA group (Additional file 1: Table S1). The change in CPM function score differed significantly among the three groups at 2 weeks after randomization (Table 2). The CPM function score increased in the strong EA group by 14.85, in the weak EA group by 4.75, and in the sham EA by 1.43; a greater increment of CPM function score was observed in the strong EA group than in the sham EA group (between-group difference 13.54; 95% CI 13.23 to 13.85; *P* < .01) and in the weak EA vs. sham EA group (3.80; 95% CI 3.45 to 4.15; *P* < .01). In addition, the strong EA group was also statistically different from the weak EA group (9.73; 95% CI 9.44 to 10.02; *P* < .01) (Table 2). As shown in Fig. 2, the mean CPM scores were similar among the three groups before treatment, with differences between true EA (strong and weak EA) and sham EA, and strong and weak EA becoming apparent after 2 weeks treatment. Moreover, at the end of week 2, VAS value, WOMAC, and all secondary outcomes (NPRS, ES, and PPI) were significantly lower in the two true EA groups than in the sham EA group (*P* < .01 for all comparisons), and strong EA was more effective in improving VAS, NPRS, and ES than weak EA (Table 2). Results of per-protocol analyses were included in the supporting information **(**Additional file 2: Table S2).

**Table 2:** Primary and secondary outcome measurements of intention-to-treat analysis during the entire study

Outcome	Strong EA	Weak EA	Sham EA	Pairwise comparison					
				Strong EA vs. sham EA		Weak EA vs. sham EA		Strong EA vs. weak EA	
				Effect size (95% CI)	*P* value	Effect size (95% CI)	*P* value	Effect size (95% CI)	*P* value
Primary outcome									
CPM, mean (SD)^a^									
Weeks 0–1	0.64 (0.16)	0.12 (0.27)	0.44 (0.24)	0 (0 to 0)	.33	0 (− 0.31 to 0.32)	0.98	0.15 (− 0.13 to 0.42)	.29
Weeks 0–2	14.85 (0.16)	4.75 (0.28)	1.43 (0.24)	13.54 (13.23 to 13.85)	< .01	3.80 (3.45 to 4.15)	< .01	9.73 (9.44 to 10.02)	< .01
VAS, mean (SD)^b^									
Weeks 0–1	− 1.34 (0.10)	− 1.52 (0.14)	0.65 (0.14)	− 0.58 (− 0.71 to − 0.44)	< .01	− 0.77 (− 0.93 to − 0.62)	< .01	0.19 (0.04 to 0.35)	.01
Weeks 0–2	− 2.97 (0.10)	− 2.75 (0.15)	1.19 (0.14)	− 1.58 (− 1.75 to − 1.4)	< .01	− 1.36 (− 1.57 to − 1.16)	< .01	− 0.22 (− 0.42 to − 0.03)	.03
WOMAC, mean (SD)^c^									
Weeks 0–1	− 13.03 (0.56)	− 13.06 (0.87)	− 3.47 (0.80)	− 9.35 (− 10.75 to − 7.96)	< .01	− 9.66 (− 11.26 to − 8.07)	< .01	0.18 (− 1.23 to 1.59)	.80
Weeks 0–2	− 20.92 (0.56)	− 20.55 (0.89)	− 8.87 (0.81)	− 11.70 (− 12.52 to − 10.89)	< .01	− 11.55 (− 12.52 to − 10.58)	< .01	− 0.15 (− 1.06 to 0.75)	.74
Secondary outcome									
NPRS, mean (SD)^c^									
Weeks 0–1	− 1.59 (0.10)	1.12 (0.14)	0.72 (0.14)	− 0.93 (− 1.1 to − 0.76)	< .01	− 0.69 (− 0.9 to − 0.49)	< .01	− 0.11 (− 0.3 to − 0.07)	.24
Weeks 0–2	− 2.97 (0.11)	2.48 (0.14)	1.40 (0.14)	− 1.55 (− 1.76 to − 1.33)	< .01	− 1.07 (− 1.31 to − 0.83)	< .01	− 0.47 (− 0.7 to − 0.24)	< .01
ES, mean (SD)^c^									
Weeks 0–1	− 1.69 (0.12)	− 1.36 (0.16)	0.71 (0.13)	− 0.91 (− 1.09 to − 0.73)	< .01	− 0.67 (− 0.88 to − 0.45)	< .01	− 0.3 (− 0.5 to − 0.1)	< .01
Weeks 0–2	− 3.28 (0.13)	− 2.56 (0.16)	1.24 (0.13)	− 1.88 (− 2.12 to − 1.65)	< .01	− 1.22 (− 1.49 to − 0.95)	< .01	− 0.67 (− 0.92 to − 0.42)	< .01
PPI, mean (SD)^c^									
Weeks 0–1	− 0.70 (0.07)	− 0.68 (0.09)	0.29 (0.09)	− 0.44 (− 0.58 to − 0.29)	< .01	− 0.45 (− 0.62 to − 0.28)	< .01	0.04 (− 0.11 to 0.19)	.59
Weeks 0–2	− 1.46 (0.07)	− 1.39 (0.09)	0.65 (0.09)	− 0.78 (− 0.96 to − 0.6)	< .01	− 0.82 (− 1.03 to − 0.6)	< .01	0.04 (− 0.16 to 0.24)	.72

*EA* electroacupuncture, *VAS* visual analog scale, *CPM* conditioned pain modulation, *WOMAC* Western Ontario and McMaster Universities Osteoarthritis Index, *NPRS* numeric pain rating scale, *ES* emotional scale, *PPI* present pain intensity

^a^Higher values indicate better status

^b^Rating scales 0 to 10, with 0 being no pain and 10 being excruciating

^c^Lower values indicate better status

During the treatment course (the first week), there was no significant difference among the three groups in the CPM value (*P* > .05 for all comparisons) (Table 2). On the other hand, compared with the sham EA group, both strong and weak EA groups showed a significant improvement in all pain-related scores of VAS, WOMAC, NPRS, ES, and PPI (*P* < .01 for all comparisons). Furthermore, no differences were observed between the strong EA group and the weak EA group in the WOMAC, NPRS, or PPI scores (*P* > .05 for all comparisons) except for the VAS and ES scores at week 1.

### Safety

During the 2-week trial, 10 participants experienced serious AEs (5 coronary heart disease, 1 stroke, 1 fracture, 1 pulmonary embolism, 1 lung infection, and 1 nephritis). All individuals were admitted to a hospital, and their conditions were considered unrelated to the study or intervention. Among 145 participants (15.2%) who received at least 1 week of strong EA treatment, 22 AEs were recorded (15 subcutaneous hemorrhage or bleeding, 7 needling pain and nausea); 72 participants (13.9%) receiving weak EA reported 10 side effects (7 subcutaneous hemorrhage and 3 needling pain or nausea); and in 75 participants (14.7%) who underwent sham EA treatment, 11 side effects were recorded (7 subcutaneous hemorrhage or bleeding and 4 needling pain).

## Discussion

In this study, participants with KOA who received strong or weak EA had significantly improved VAS, WOMAC, NPRS, ES, and PPI but not CPM after 1 week of treatment than those who received sham EA. Additionally, strong EA and weak EA further increased CPM value than sham EA, and the effect of strong EA was better than weak EA after 2 weeks of treatment. Therefore, at least 2 weeks duration is necessary for EA to exert a clinical effect on KOA, with strong EA being the most effective in alleviating pain intensity and increased CPM value of KOA, thus preventing the development of chronic pain.

There is a growing body of evidence suggesting that CPM may be an important predictor of chronic pain and assessor of therapeutic effect [31]. CPM is a psychophysical experimental measure of the endogenous pain inhibitory pathway in humans [32], a phenomenon in which one noxious stimulus prevents the pain generated by another noxious stimulus [33]. Less-efficient CPM have been reported in people with chronic pain, and impaired CPM might have a role in the development and maintenance of chronic pain [34–36].

In humans, to our knowledge, ours is the first study to assess the efficacy of EA in improving the CPM in patients with KOA. Since CPM modulates the transmission of nociceptive signals involving the periaqueductal gray, rostral ventromedial medulla, and subnucleus reticularis dorsalis [37, 38], the strength of CPM may reflect the function of the endogenous pain inhibitory system. Endogenous opioid peptides, GABA, serotonin, and the noradrenergic system are involved in the regulation of descending pain control [39–41]. Our results suggested that 1 week of strong or weak EA treatment is not sufficient for repairing CPM and that at least 2 weeks of EA is needed to enhance the synthesis of endogenous analgesic neurotransmitters and strengthen the function of the endogenous pain inhibitory system. Thus, although EA has an immediate effect on pain intensity, a cumulative effect is required for EA to repair CPM value. In addition, EA intervention was well tolerated, with only a 7.2% attrition rate. Our low dropout rate and low incidence of adverse events highlight the feasibility of EA in treating KOA.

CPM is specifically activated by peripheral A and/or C fibers. Weak EA with low-intensity current (less than 1 mA) has been demonstrated to mainly stimulate large fibers (Aβ fibers), which may not activate CPM function [8]. Instead, this harmless stimulation may only inspire an analgesic effect through the spinal mechanism of gate control theory [9]. In contrast, strong EA with high-intensity current (more than 2 mA) stimulates thin fibers (Aδ and/or C fibers) and may activate CPM function [10]. In this study, we mainly discussed the influence of EA intervention on KOA patients based on previous findings of CPM involvement in the analgesic mechanism of acupuncture [42, 43]. In our study, CPM (the DNIC-like function in human) was assessed by measuring the rate of change in VAS before and after nociceptive cold water stimulus. All participants in the three groups had significant improvement in CPM function after 2 weeks treatment. Participants who received strong or weak EA had significantly better CPM function than those who received sham EA, excluding the placebo effect of sham EA. As the previous study [44] showed that true EA was better than the placebo effect of sham EA but the study was not an RCT, ours is the first study in RCT to prove that the effect of true EA (strong EA and weak EA) is better than the placebo effect of sham EA.

Strong EA had a better effect on repairing CPM function than weak EA, suggesting that high-intensity current (the maximum tolerable intensity of current) was the ideal electric current intensity of EA that could prevent the development and maintenance of chronic pain of KOA. Our results are in line with the previous study that high intensity (to tolerance but subnoxious) of EA is better than low intensity (strong but comfortable) of EA in reducing experimental pain in healthy human volunteers [11]. Our results are also the first to show that strong EA was better than weak or sham EA in alleviating pain intensity and inhibiting chronic pain of KOA.

ITT analysis indicated that there was no significant difference in CPM improvement between sham EA and weak EA/strong EA at the end of week 1; the positive effect of strong EA on CPM function seems to become apparent beginning in week 2. These findings were further confirmed by the PP analysis. All recruited participants were of Han ethnicity, many of whom knew about or had been exposed to acupuncture. The pain in participants with KOA who hold a positive attitude toward acupuncture is more likely to improve [45]. There are some limitations in our study. First, although the participants were not aware of which type of EA they received, it was not possible to blind the acupuncturists who administered EA. Participant expectations and their existing positive attitude toward acupuncture, acupuncturists’ confidence in treatment, and interaction between participant and acupuncturist may have influenced the outcomes to some extent. Second, the inability to directly assess the correlation between treatment expectancy and the specific effects of EA on CPM function prevented the determination of the magnitude of these different effects. In addition, imaging studies, such as X-ray, were not performed. Therefore, we could not conclude whether and how the severity of disease affected the treatment response. Finally, the duration of EA intervention was only 2 weeks because our goal was to evaluate the short-term effect of EA. Thus, future studies are required to explore the long-lasting effect of EA in improving CPM function in patients with KOA.

## Conclusions

In conclusion, strong or weak EA should be administered for at least 2 weeks to exert a clinically important effect on KOA. Strong EA was better than weak or sham EA in reducing VAS and improving CPM function and was the most effective in alleviating pain intensity and the development of chronic pain of KOA patients. This study will not only increase our knowledge on the effects of strong or weak EA on treating chronic pain in patients with KOA, but also may help acupuncturist to choose the optimal intensity of EA and promote the clinical effect of EA analgesia. The selection of strong EA may be considered for inclusion in clinical guidelines for EA in the treatment of chronic pain of KOA. Moreover, the strong EA should be effective for other chronic pain diseases other than KOA, which will be very helpful in solving opioid overdose.

## Additional files

Additional file 1: **Table S1.** Descriptive statistics of mean (SD) scores on outcome measures over time according to group. (DOCX 18 kb)

Additional file 2: **Table S2.** Primary and secondary outcome measurements of per-protocol analysis during the entire study. (DOCX 20 kb)

## Abbreviations

BMI: Body mass index
CPM: Conditioned pain modulation
DNIC: Diffuse noxious inhibitory controls
EA: Electroacupuncture
ES: Emotional scale
ITT: Intention-to-treat
KOA: Knee osteoarthritis
MCID: Minimal clinically important difference
NPRS: Numeric pain rating scale
OA: Osteoarthritis
PP: Per-protocol
PPI: Present pain intensity
RCT: Randomized controlled trial
VAS: Visual analog scale
WOMAC: Western Ontario and McMaster Universities Osteoarthritis Index
