# Supplementary material for: Effects of intensity of electroacupuncture on chronic pain in patients with knee osteoarthritis: a randomized controlled trial
Source: Arthritis Res Ther. 2019 May 14;21:120. doi: 10.1186/s13075-019-1899-6 (PMC6518678; doi:10.1186/s13075-019-1899-6)
Supplement: Supplementary file 1 — Table S1. Descriptive statistics of mean (SD) scores on outcome measures over time according to group. (DOCX 18 kb) [file 13075_2019_1899_MOESM1_ESM.docx]

**Table S1.** Descriptive statistics of mean (SD) scores on outcome measures over time according to group.

| **Outcome** | **Strong EA** | | **Weak EA** | | | | **Sham EA** | | |
| --- | --- | --- | --- | --- | --- | --- | --- | --- | --- |
| **Primary outcome** |  | | |  | | | |  | |
| ***CPM, mean (SD)*** ^a^ | |  | | |  | | | |  |
| Week 0 (Baseline) | 9.49 (1.90) | | 9.86 (2.50) | | | 9.46 (2.05) | | | |
| Week 1 | 10.13 (0.97) | | 9.99 (1.02) | | | 9.90 (1.00) | | | |
| Week 2 | 24.34 (0.87) | | 14.61 (0.79) | | | 10.89(1.07) | | | |
| ***VAS, mean (SD)*** ^b^ | |  | |  | | | | |  |
| Week 0 (Baseline) | 7.50 (0.91) | | 7.54 (0.99) | | | 7.23 (0.97) | | | |
| Week 1 | 6.16 (0.86) | | 6.02 (0.86) | | | 6.58 (0.83) | | | |
| Week 2 | 4.53 (0.84) | | 4.79 (0.66) | | | 6.04 (0.75) | | | |
| ***WOMAC, mean (SD)*** ^c^ | |  | | |  | | | |  |
| Week 0 (Baseline) | 31.86 (4.67) | | 31.82 (5.96) | | | 31.80 (5.49) | | | |
| Week 1 | 18.83 (5.98) | | 18.76 (5.98) | | | 28.33 (6.06) | | | |
| Week 2 | 10.94 (2.93) | | 11.27 (2.79) | | | 22.93 (2.04) | | | |
| **Secondary outcome** | |  | | |  | | | |  |
| ***NPRS, mean (SD)*** ^c^ | |  | | |  | | | |  |
| Week 0 (Baseline) | 6.83 (0.96) | | 6.69 (0.94) | | | 6.91 (1.02) | | | |
| Week 1 | 5.25 (0.82) | | 5.28 (0.75) | | | 6.19 (0.80) | | | |
| Week 2 | 3.87 (0.87) | | 4.22 (0.72) | | | 5.51 (0.67) | | | |
| ***ES, mean (SD)*** ^c^ | |  | | |  | | | |  |
| Week 0 (Baseline) | 6.76 (1.16) | | 6.61 (1.03) | | | 6.57 (0.92) | | | |
| Week 1 | 5.07 (1.00) | | 5.25 (0.83) | | | 5.87 (0.79) | | | |
| Week 2 | 3.48 (0.98) | | 4.05 (0.93) | | | 5.34 (0.67) | | | |
| ***PPI, mean (SD)*** ^c^ | |  | | |  | | | |  |
| Week 0 (Baseline) | 3.46 (0.59) | | 3.36 (0·54) | | | 3.49 (0.53) | | | |
| Week 1 | 2.75 (0.58) | | 2.68 (0.55) | | | 3.20 (0.52) | | | |
| Week 2 | 1.99 (0.65) | | 1.97 (0.50) | | | 2.85 (0.55) | | | |

Abbreviations: EA, electroacupuncture; VAS, visual analog scale; CPM, conditioned pain modulation; WOMAC, Western Ontario and McMaster Universities Osteoarthritis Index; NPRS, numeric pain rating scale; ES, emotional scale; PPI, present pain intensity.

^a^ Higher values indicate better status.

^b^ Rating scale 0 to 10, with 0 being no pain and 10 being excruciating.

^c^ Lower values indicate better status.
