# Supplementary material for: Effects of intensity of electroacupuncture on chronic pain in patients with knee osteoarthritis: a randomized controlled trial
Source: Arthritis Res Ther. 2019 May 14;21:120. doi: 10.1186/s13075-019-1899-6 (PMC6518678; doi:10.1186/s13075-019-1899-6)
Supplement: Supplementary file 2 — Table S2. Primary and secondary outcome measurements of per-protocol analysis during the entire study. (DOCX 20 kb) [file 13075_2019_1899_MOESM2_ESM.docx]

**Table S2.** Primary and Secondary Outcome Measurements of Per-protocol Analysis During the Entire Study.

| **Outcome** | **Pairwise Comparison** | | | | | | | | | |
| --- | --- | --- | --- | --- | --- | --- | --- | --- | --- | --- |
|  | **Strong EA vs. Sham EA** | | | | **Weak EA vs. Sham EA** | | | | **Strong EA vs. Weak EA** | |
|  | **Effect Size (95% CI)** | | | **P value** | **Effect Size (95% CI)** | | | **P value** | **Effect Size (95% CI)** | **P value** |
| **Primary outcome** | | | | | | | | |  |  |
| ***CPM, mean (SD)*** ^a^ |  |  |  | | |  |  |  |  |  |
| Week 0-1 | 0.36 (0.08 to 0.63) | | | .01 | -0.19 (-0.12 to 0.51) | | | 0.23 | 0.15 (-0.13 to 0.42) | .29 |
| Week 0-2 | 13.54 (13.23 to 13.85) | | | <.01 | 3.80 (3.45 to 4.15) | | | <.01 | 9.73 (9.47 to 10.00) | <.01 |
| ***VAS, mean (SD)*** ^b^ |  |  |  | | |  |  |  |  |  |
| Week 0-1 | -0.65 (-0.79 to -0.51) | | | <.01 | -0.83 (-0.99 to -0.67) | | | <.01 | 0.19 (0.04 to 0.35) | .01 |
| Week 0-2 | -1.58 (-1.75 to -1.4) | | | <.01 | -1.36 (-1.57 to -1.16) | | | <.01 | -0.25 (-0.44 to -0.07) | .01 |
| ***WOMAC, mean (SD)*** ^c^ |  |  |  | | |  |  |  |  |  |
| Week 0-1 | -9.47 (-10.83 to -8.10) | | | <.01 | -9.79 (-11.37 to -8.20) | | | <.01 | 0.18 (-1.23 to 1.59) | .80 |
| Week 0-2 | -11.70 (-12.52 to -10.89) | | | <.01 | -11.55 (-12.52 to -10.58) | | | <.01 | -0.16 (-0.99 to 0.75) | .70 |
| **Secondary outcome** |  |  |  | | |  |  |  |  |  |
| ***NPRS, mean (SD)*** ^c^ |  |  |  | | |  |  |  |  |  |
| Week 0-1 | -0.98 (-1.15 to -0.82) | | | <.01 | -0.82 (-1.01 to -0.63) | | | <.01 | -0.11 (-0.30 to -0.07) | .24 |
| Week 0-2 | -1.55 (-1.76 to -1.33) | | | <.01 | -1.07 (-1.31 to -0.83) | | | <.01 | -0.48 (-0.70 to -0.27) | <.01 |
| ***ES, mean (SD)*** ^c^ |  |  |  | | |  |  |  |  |  |
| Week 0-1 | -0.94 (-1.11 to -0.77) | | | <.01 | -0.66 (-0.86 to -0.46) | | | <.01 | -0.30 (-0.50 to -0.1) | <.01 |
| Week 0-2 | -1.88 (-2.12 to -1.65) | | | <.01 | -1.22 (-1.49 to -0.95) | | | <.01 | -0.67 (-0.91 to -0.44) | <.01 |
| ***PPI, mean (SD)*** ^c^ |  |  |  | | |  |  |  |  |  |
| Week 0-1 | -0.57 (-0.72 to -0.42) | | | <.01 | -0.56 (-0.74 to -0.39) | | | <.01 | 0.04 (-0.11 to 0.19) | .59 |
| Week 0-2 | -0.78 (-0.96 to -0.6) | | | <.01 | -0.82 (-1.03 to -0.6) | | | <.01 | 0.05 (-0.13 to 0.24) | .58 |

Abbreviations: EA, electroacupuncture; VAS, visual analog scale; CPM, conditioned pain modulation; WOMAC, Western Ontario and McMaster Universities Osteoarthritis Index; NPRS, numeric pain rating scale; ES, emotional scale; PPI, present pain intensity.

^a^ Higher values indicate better status.

^b^ Rating scale 0 to 10, with 0 being no pain and 10 being excruciating.

^c^ Lower values indicate better status.
